# Genetics of Behçet's Disease: Functional Genetic Analysis and Estimating Disease Heritability

**DOI:** 10.3389/fmed.2021.625710

**Published:** 2021-02-12

**Authors:** Lourdes Ortiz-Fernández, Amr H. Sawalha

**Affiliations:** ^1^Division of Rheumatology, Department of Pediatrics, University of Pittsburgh School of Medicine, Pittsburgh, PA, United States; ^2^Division of Rheumatology and Clinical Immunology, Department of Medicine, University of Pittsburgh School of Medicine, Pittsburgh, PA, United States; ^3^Lupus Center of Excellence, University of Pittsburgh School of Medicine, Pittsburgh, PA, United States; ^4^Department of Immunology, University of Pittsburgh School of Medicine, Pittsburgh, PA, United States

**Keywords:** Behçet's disease, genetics, heritability, genetic risk score, epigenetic

## Abstract

Behçet's disease is a chronic multisystemic inflammatory disorder characterized by recurrent oral and genital ulcers. Although its etiology remains unclear, it is thought that both genetic and environmental factors contribute to the onset and progression of Behçet's disease. Here, we provide an updated view of the genetic landscape and architecture of Behçet's disease. Large-scale genetic studies performed to date revealed 21 genetic susceptibility loci associated with the disease at a GWAS level of significance (*p*-value = 5 × 10^−8^). We performed epigenetic pattern enrichment analysis in Behçet's disease associated loci, providing new insights into the molecular mechanisms underlying its pathophysiology. Our data suggest the crucial involvement of several immune cell types, including natural killer cells, monocytes, and B cells in the pathogenesis of the disease. Pathway enrichment analysis identified important biological processes involved. Using large-scale genetic data available from ~200 immune-related loci (Immunochip), we estimate Behçet's disease heritability to be at least 16%. We further used the same approach to estimate the heritability explained by the known Behçet's disease-associated loci, suggesting that they explain ~ 60% of the genetic component underlying Behçet's disease. These results indicate a significant role of non-genetic factors in causing Behçet's disease and that additional genetic variation influencing the risk of Behçet's disease remains to be identified. Finally, we calculated a cumulative genetic risk score across populations reinforcing the link between geographic variations in disease prevalence with its genetic component.

## Introduction

Behçet's disease is a chronic inflammatory disease with a relapsing-remitting course characterized by recurrent oral and genital ulcers. It is a debilitating systemic vasculitis that can affect the eyes, skin, blood vessels, central nervous system, and gastrointestinal tract, leading to a wide range of clinical manifestations (1, 2). Behçet's disease onset is typically in the third or fourth decade of life, although younger adults and children can be affected. Both genders are equally affected, however, a more severe course of the disease has been described in men and in the young (3). Behçet's disease patients have been diagnosed worldwide, although its highest prevalence coincides with the countries stretching from East Asia to the Mediterranean region along the ancient trading route “silk road” (4, 5).

Currently, the pathogenesis of Behçet's disease remains unclear; however, the consensus is that the disease is triggered by exposure to environmental factors, such as infectious agents and others, in individuals with a genetic susceptibility background (6). The role that infections might play in Behçet's disease pathogenesis has long been interrogated. Poor oral health is associated with a more severe course of the disease and the results of recent studies investigating the oral microbiome revealed that the salivary microbial community in Behçet's disease is less diverse than in healthy controls (7, 8).

The contribution of genetic factors to the pathogenesis of Behçet's disease is strongly supported. In the early 1970's, the identification of the human leukocyte antigen (HLA) region as the first susceptibility genetic region for Behçet's disease was reported. By using serological methods Ohno and collaborators detected the association of HLA-B51 with Behçet's disease (9). Almost fifty years later, the challenging task of unraveling the genetic architecture of Behçet's disease is still ongoing.

Here we examined our current knowledge of the genetic basis of Behçet's disease paying special attention to the findings obtained in the large-scale genetic studies performed over the recent years. We further used these genetic information to yield new insights into the genetic architecture of this disorder. We first summarized the loci that have been previously associated with Behçet's disease. Next, we used bioinformatic tools and publicly available data to perform functional annotation and enrichment analyses, to gain insights into the molecular mechanisms driven by the genetic associations in Behçet's disease. We pinpoint key cell types and molecular pathways that may be involved in the disease pathophysiology. Finally, we estimated the heritability of Behçet's disease for the first time and calculated a cumulative genetic risk score across various populations for this disease.

## The Contribution of Large-Scale Genetic Studies in Deciphering the Complex Genetic Architecture of Behçet's Disease

The genetic component of Behçet's disease was initially interrogated by candidate gene association studies, which were mainly focused on investigating genes with known associations with other immune-mediated diseases. Over several decades, investigators have conducted multiple candidate gene studies analyzing a myriad of candidate genes in different populations (10). This strategy had significant limitations, and the associations identified by candidate gene studies were mostly observed in single cohorts, and generally reported weak genetic effects that were difficult to replicate in subsequent studies.

Large-scale genetic studies, and genome-wide association studies (GWAS) in particular, have provided a powerful unbiased approach to investigate the genetics of complex diseases allowing for the identification of thousands of genetic variants associated with hundreds of human complex traits and diseases (11). Large-scale genetic studies performed in Behçet's disease are summarize in Table 1.

**Table 1:** Summary of the large-scale genetic studies performed in Behçet's disease.

**Year**	**First Author**	**Discovery stage**				**Replication stage**				**References**
		**Cohort**		**Genotyping**	**Imputation**	**Cohort**		**Genotyping**	**Imputation**	
		**Ethnicity**	**Case/Control**			**Ethnicity**	**Case/Control**			
2009	Fei Y	Turkish	152/172	Affymetrix GeneChip® Human Mapping 500 K Array^$^	NO					(12)
2010	Mizuki N	Japanese	612/740	Affymetrix GeneChip® Human Mapping 500 K Array	NO	Korean	119/140	TaqMan SNP genotyping assays	NO	(13)
						Turkish	1,215/1,278	Data obtained from (14)	NO	
2010	Remmers EF	Turkish	1,321/1,336	HumanCNV370-Duo v1.0 and Human CNV370-Quad v3.0	NO	Turkish	111/225	TaqMan SNP genotyping assays		(14)
						Middle Eastern Arab	189/163			
						Greek	107/84			
						UK European descent	120/119			
						Korean	77/52			
						Japanese	612/740	Data obtained from (13)	NO	
2012	Lee YJ	Korean	379/800	Affymetrix genome-wide human single nucleotide polymorphism (SNP) array 6.0	NO	Japanese	363/272	TaqMan SNP genotyping assays	NO	(15)
2012	Hou S	Han Chinese	149/951	Affymetrix genome-wide human single nucleotide polymorphism (SNP) array 6.0	NO	Han Chinese	554/1,159	iPLEX assays (TOF-MS, Sequenom)	NO	(16)
2013	Kirino Y	Turkish	1,209/1,278^^†^^	HumanCNV370-Duo v1.0 and Human CNV370-Quad v3.0	YES	Turkish	838/630	iPLEX assays (TOF-MS, Sequenom) and TaqMan SNP genotyping assays	NO	(17)
						Japanese	612/740	Data obtained from (13)	NO	
2013	Hughes T	Turkish	503/504	Infinium ImmunoArray-24 V.1.0 BeadChip	HLA imputation					(18)
		Italian	144/1,270							
2014	Ombrello MJ	Turkish	1,190/1,257	Infinium Human CNV370 arrays (Illumina)	HLA imputation					(19)
2015	Xavier JM	Iranian	292/294	Affymetrix genome-wide human single nucleotide polymorphism (SNP) array 6.0^$^	NO	Iranian	684/532	iPLEX assays (TOF-MS, Sequenom)	NO	(20)
						Turkish	1,215/1,278	Data obtained from (17)	YES	
2015	Kappen JH	Multi-ethnic^*^	369/5,843	Illumina HumanHap 610K and/or 660 K arrays	NO	Western European	82/98	TaqMan SNP genotyping assays	NO	(21)
						Turkish		Data obtained from (17)		
2016	Ortiz-Fernandez L	Spanish	286/1,517	Infinium ImmunoArray-24 V.1.0 BeadChip	HLA imputation	Spanish	130/600	TaqMan SNP genotyping assays	NO	(22)
2017	Takeuchi M	Turkish	2,014/1,826	Infinium ImmunoArray-24 V.1.0 BeadChip	YES	Iranian	969/826	TaqMan SNP genotyping assays		(23)
						Japanese	608/737	TaqMan SNP genotyping assays	YES	
2020	Ortiz-Fernandez L	Turkish	2,532/1,977	Infinium ImmunoArray-24 V.1.0 and V.2.0 BeadChip	YES					(24)
		Spanish	278/1,517							
		Italian	144/1,270							
		Korean	200/200							
		Tunisian	136/186							
		Japanese	120/218							
		Western European	67/599							

$ *DNA pooling technology was used for the array genotyping. A subsequent individual genotyping for the candidate genes discovered was performed with TaqMan SNP genotyping assays*.

† *Genotyping data from (14)*.

* *Includes patients from 18 different geographic origins*.

In 2009, Fei et al. performed the first GWAS in Behçet's disease in a Turkish population using a DNA pooling approach (12). Although no loci were identified at a GWAS level of significance, this study constituted a milestone in studying the genetics of Behçet's disease, and identified novel genetic susceptibility loci for the disease including genetic effects that were replicated in subsequent studies and in other populations. The main limitation of this work was the relatively low sample size to achieve the established threshold of significance for these studies (*p*-value = 5 × 10^−8^). One year later, GWAS studies including larger cohorts were performed revealing two loci associated with Behçet's disease at GWAS level of significance: interleukin 23 receptor (*IL23R*) and the interleukin 10 (*IL10*) (Tables 1, 2) (13, 14). Both loci have been replicated in multiple populations and are considered established susceptibility factors for Behçet's disease (22, 23, 25–32). The development of faster and more accurate imputation methods allowed the evaluation and statistical interrogation of additional genetic variants in complex diseases using pre-existing genetic data. In this regard, Kirino et al. detected three new associated loci after imputing from the genetic data obtained from a previous GWAS, and provided important insight into the pathogenesis of Behçet's disease including the possibility of genetic interaction between HLA-B^*^51 and *ERAP1* (14, 17) (Tables 1, 2).

**Table 2:** Genetic susceptibility loci associated with Behçet's disease at GWAS level of significance (*p* < 5 × 10^−8^).

**Reported gene**	**Chr**	**SNPs**	**Candidate genes**	**References**
*IL12RB2, IL23R*	1	rs10889664, rs6660226, rs1495965, **rs924080**		(13, 14, 22, 24)
*IL10*	1	**rs1518111**, rs1518110, rs3024490, rs1800871		(13, 14, 23, 24)
*IL1A, IL1B*	2	**rs3783550**	*IL37*	(23)
*TFCP2L1*	2	**rs17006292**		(16)
*STAT4*	2	**rs7574070**, rs897200		(16, 17)
*CCR1, CCR3*	3	**rs7616215**, rs2087726	*CCR2, RTP3, FYCO1*	(17, 24)
*IL12A-AS1, IL12A*	3	rs76830965, **rs17753641**, rs17810546, rs1874886	*SCHIP1, IQCJ-SCHIP1*	(21–24)
*ERAP1*	5	**rs17482078**	*CAST*	(17)
*HLA-A*	6	rs9260997, rs112166594, **rs114854070**	*HLA-F, RNF39, TRIM31, PPP1R11*	(14, 18, 19)
*HLA-B, MICA*	6	rs4959053, rs4947296, rs79556279, rs9266490, rs1050502, rs2848713, rs7770216, rs2442736, **rs116799036**	*POU5F1*	(13–23)
*HLA-C*	6	**rs12525170**		(18)
*IFNGR1*	6	**rs4896243**		(24)
*RIPK2*	8	**rs2230801**		(23)
*Intergenic LNCAROD/DKK1*	10	**rs1660760**		(24)
*ADO, EGR2*	10	**rs224127**, rs12220700		(23, 24)
*Intergenic JRKL/ CNTN5*	11	**rs2848479**		(22)
*KLRC4*	12	**rs2617170**	*KLRC3, KLRC2, KLRC1, KLRK1*	(17)
*LACC1*	13	rs2121034, **rs2121033**	*CCDC122*	(23, 24)
*IRF8*	16	**rs7203487**		(23)
*IRF8*	16	**rs11117433**		(23)
*FUT2*	19	**rs681343**	*RASIP1, IZUMO1, NTN5*	(20)
*Intergenic CEBPB/PTPN1*	20	**rs913678**		(23)

*Lead SNPs (or the most significant SNP in each genetic region in the largest single cohort for each associated locus) are highlighted in bold. Candidate genes suggested by functional annotation are also included*.

The development of the Immunochip array represented another landmark in the efforts to study the genetics of immune-mediated diseases. The Immunichip is a custom array from Illumina designed to perform fine-mapping of almost 200 genetic loci relevant to multiple different immune-mediated disorders. This cost-effective genotyping platform was demonstrated to be a successful strategy in revealing and fine-mapping genetic associations in multitude of diseases (33). Several Immunochip studies have been performed, including the largest and most diverse genetic study reported to date in Behçet's disease in which 3,477 patients and 5,967 healthy individuals from seven different populations were analyzed (24). In this recent work, eight genetic susceptibility loci were found to be associated with Behçet's disease at a GWAS level of significance, two of which corresponded with novel genetic susceptibility loci in interferon gamma receptor 1 (*IFNGR1*) and the intergenic regions between *LNCAROD*, lncRNA activating regulator of DKK, and *DKK1*, dickkopf WNT signaling pathway inhibitor 1 (24).

The recent advances in the design of the genotyping arrays as well as in the statistical genetics and imputation algorithms have also proven successful in terms of analyzing the complex HLA region. The association of the classical allele HLA-B^*^51 is well-established and is considered the strongest genetic risk factor for Behçet's disease. More recently, a robust genetic association between a genetic variant between *HLA-B* and *MICA* was suggested to explain the association with HLA-B^*^51 in Behçet's disease. However, whether the classical allele HLA-B^*^51 or the *HLA-B*/*MICA* intergenic locus is causal in Behçet's disease requires further investigation (18, 19, 26, 34, 35). It is also likely that a haplotype effect that includes both coding and non-coding genetic variants within the HLA-B region is involved in Behçet's disease. In addition, dense genotyping followed by imputation have revealed several independent genetic effects within the complex HLA region in Behçet's disease, including in *HLA-A* and *HLA-C* (18, 19, 22, 23).

Considering all above, large-scale genetic studies have been the main contributors to our current knowledge of the genetic component of Behçet's disease. To date, a total of 21 genetic susceptibility loci for Behçet's disease that surpass the threshold for genome-wide level of significance have been discovered. These loci are summarized in Table 2 and will be subjected to additional functional analysis below.

## From Statistics to the Function

The primary goal of identifying the loci that contribute to Behçet's disease is to better understand the molecular mechanisms underlying its pathophysiology, which might result in the identification of novel drug targets. However, since most of the Behçet's disease-associated polymorphisms reside in non-coding regions of the genome, linking the statistical genetic associations to the molecular functional consequences remains a challenge. Non-coding SNPs might influence the disease by disrupting regulatory elements, and although genetic variants are inherited identically across tissues, allele-specific regulatory effects might be cell type-specific. Over the last years, new bioinformatic approaches and analysis methods have allowed the integration of large-scale genetic studies with functional genomics datasets (36). In this context, cells types that are functionally relevant in the disease pathogenesis can be inferred by using methods that test for enrichment of disease-risk variants in genomic annotations.

As the cell types involved in the molecular mechanisms driving Behçet's disease pathology are not yet completely elucidated, here we aimed to detect the specific cell subsets that are more likely to be altered by the disease-associated variants. We carried out a regulatory enrichment analysis in which we checked if the presence of epigenetic marks was more frequent than expected by chance at Behçet's disease susceptibility loci (lead SNPs highlighted in bold in Table 2 were included in this analysis). We used GenomeRunner software (37) to analyze seven different histone marks (H3K4me1, H3K4me2, H3K4me3, H3K9ac, H3K27ac, H3K27me3, and H3K9me3) across 127 reference epigenomes obtained from the Encyclopedia of DNA Elements (ENCODE) and the Roadmap Epigenomics Consortium projects (38, 39). We selected the processed gapped peak annotation panels (38) and all common SNPs from dbSNP v142 were used as background to control for functional enrichments occurring by chance. The False Discovery Rate (FDR) calculation were applied to correct the enrichment *p*-values for multiple testing (FDR *p*-values < 0.05 were considered significant). Regarding the selected histone marks, H3K4me1 is associated with enhancers and regions downstream of transcription factor binding sites, and H3K4me2 has been related with both promoters and enhancers. H3K4me3 marks active promoters. Acetylation in H3K9 and H3K27 has been associated with transcriptional initiation and open chromatin structure. Finally, H3K27me3 marks silenced promoters and H3K9me3 has been associated with repressive heterochromatic state (38, 39).

**Figure 1:**
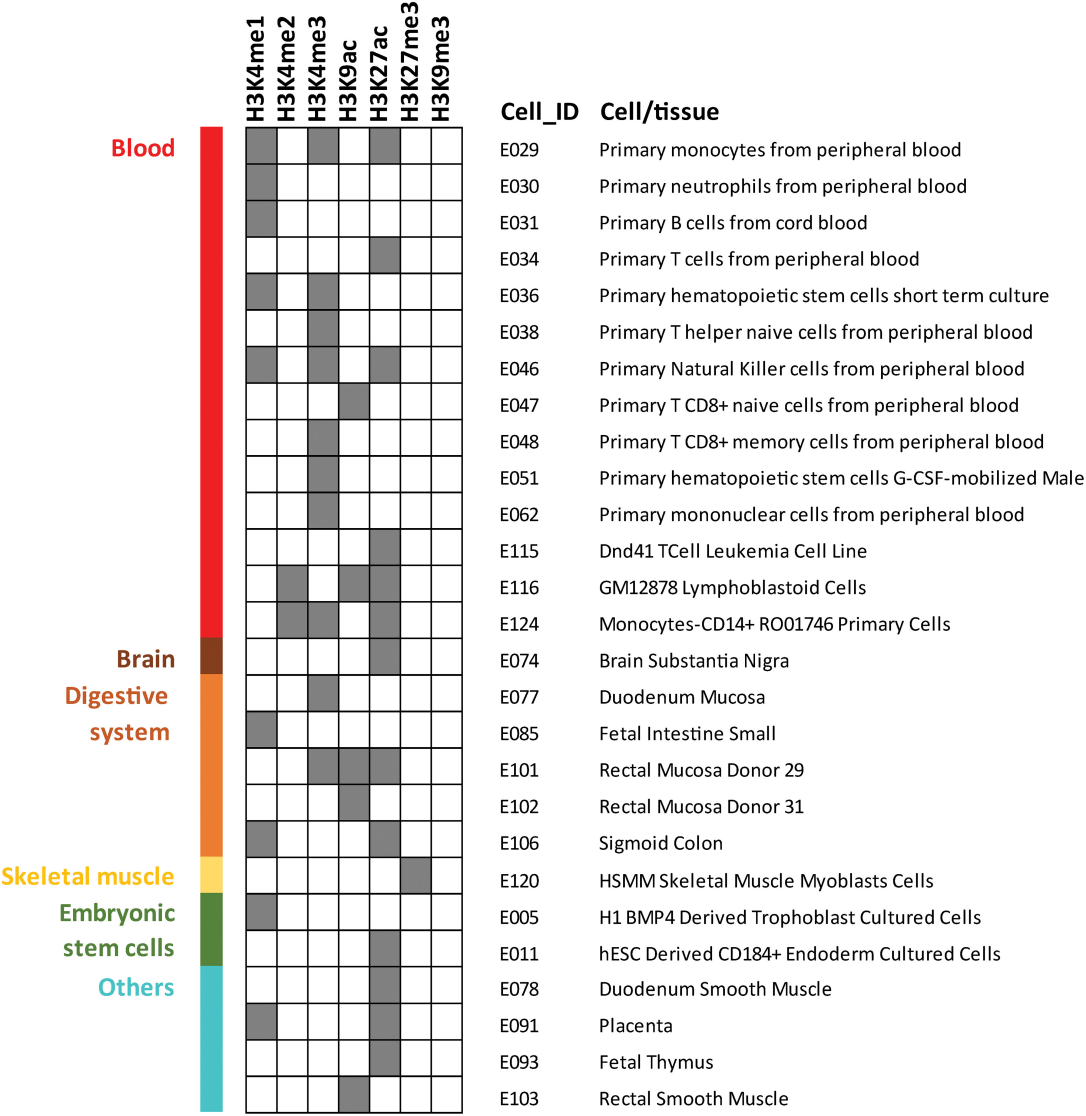
Histone marks enrichment analysis among genetic susceptibility loci previously reported at GWAS level of significance (*p*-value <5 × 10^−8^) for Behçet's disease. GenomeRunner was used to perform the enrichment of seven histone marks in 127 cell and tissue types included in Roadmap Epigenomics Consortium and the Encyclopedia of DNA Elements (ENCODE). Dark gray color squares denote statistical significance (corrected *p*-value < 0.05). Only the 27 cell and tissue types for which significant enrichment was identified for at least one histone mark are displayed.

The results of this analysis revealed 40 significantly overrepresented histone marks in 27 cell types/tissues, which are illustrated in Figure 1. A complete list of the results is shown in Supplementary Table 1. Most epigenetic mark enrichment patterns were observed in blood cells which include a wide repertoire of immune cells. In addition, the histone marks detected represent cell activation. Therefore, the histone enrichment findings in the reported Behçet's disease-susceptibility loci reflected a robust immunological signature which is consistent with our understanding of the disease. Specifically, the stronger enrichment patterns were observed in natural killer (NK): H3K27ac (*p*-value = 3.71 × 10^−6^), H3K4me1 (*p*-value = 3.35 × 10^−2^) and H3K4me3 (*p*-value = 1.39 × 10^−2^); in a lymphoblastoid cell line (GM12878): H3K9ac (*p*-value = 9.87 × 10^−5^), H3K27ac (*p*-value = 6.72 × 10^−3^) and H3K4me2 (*p*-value = 2.47 × 10^−2^); and in monocytes, including primary monocytes: H3K27ac (*p*-value = 5.65 × 10^−4^), H3K4me3 (*p*-value = 2.18 × 10^−3^) and H3K4me1 (*p*-value = 1.39 × 10^−2^) and monocytes-CD14+: H3K4me2 (*p*-value = 7.65 × 10^−3^), H3K4me3 (*p*-value = 1.55 × 10^−2^) and H3K27ac (*p*-value = 3.67 × 10^−2^). These results pinpoint to NK, monocytes, and B cells as important cell types likely implicated in the pathophysiology of Behçet's disease and indicate that further investigation to elucidate their role should be conducted. In addition, the enrichments detected in T cells are remarkable as well: H3K9ac (*p*-value = 4.91 × 10^−5^) in primary naïve CD8+ T cells and H3K27ac (*p*-value = 5.56 × 10^−4^) in primary T cells. These findings reinforce the known involvement of T cells in the pathogenesis of Behçet's disease (40). Finally, additional epigenetic mark co-localizations were also observed in other immune cells that have been suggested to be implicated in the disease, such as other subsets of T cells and neutrophils. Specifically, neutrophils have been postulated to be involved in the immunologic dysfunction in Behçet's disease but the mechanism underlying their hyperfunction remains to be explored (41). Further functional studies to decipher the immunological mechanisms involved in the development and progression of Behçet's disease will help in better management of this disease. Our findings also revealed enrichment patterns across non-immune related tissues, such as brain and digestive tissues, which is in agreement with known organ involvement in Behçet's disease.

Identifying the causal genes responsible for the genetic association signals detected in genetic studies represents another challenge for translating genetic findings into disease mechanisms and new treatments. This task might be relatively more straightforward for coding variants that directly disrupt the structure of a protein. However, as stated earlier, most of the disease-associated genetic variants in Behçet's disease are in non-coding regions of the genome which makes it difficult to link them to a candidate causal gene. Genes located near the risk variants are the most immediate obvious candidates for further investigation but the gene nearest to the polymorphism might not necessarily be relevant or causal.

We evaluated the potential causality of the Behçet's disease-associated genetic signals to identify candidate causal genes. We first explored expression quantitative trait loci (eQTL) across different human tissues by querying Genotype-Tissue Expression (GTEx) project (42). We found that 72.7% of Behçet's disease associated genetic variants have been identified to alter expression of at least one gene (Supplementary Table 2). Next, under the assumption that chromatin architecture may influence transcriptional regulation, we investigated if interactions via long-range chromatin loops between Behçet's disease risk variants and distal elements exist. We used the Capture HiC Plotter webtool to detect and visualize physical chromatin interactions between Behçet's disease-associated variants and gene promoter regions across multiple cell types (43). Most of inspected signals showed multiple interactions as shown in Supplementary Table 2. Of particular interest are those interactions with the promoters of genes whose expression levels were also altered by the same genetic variant in our eQTL analysis. These genes are highlighted in bold in Supplementary Table 2 and are listed in Table 2. Further investigation should be performed to assess the potential role these identified candidate genes might play in the pathogenesis of Behçet's disease.

**Figure 2:**
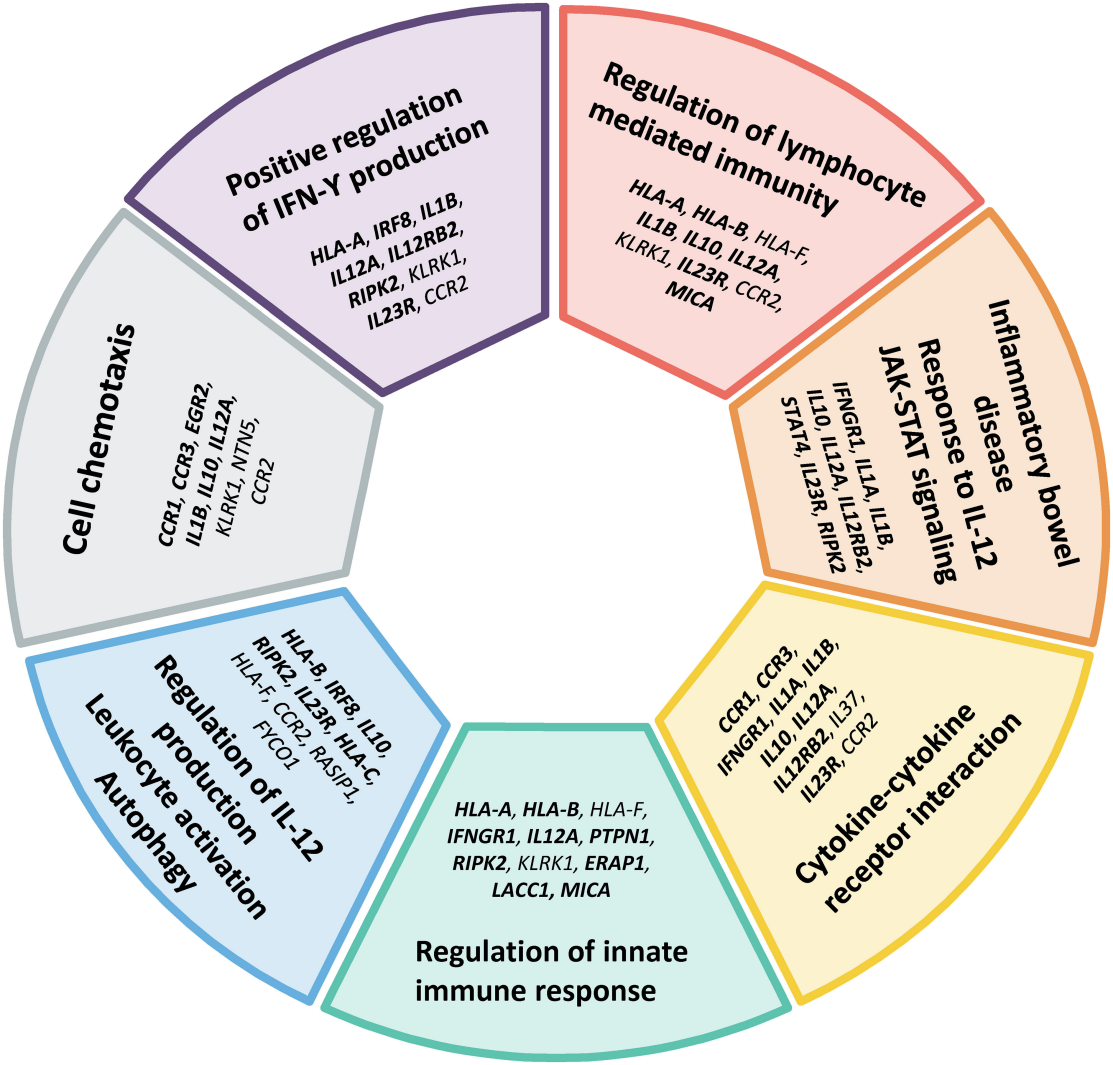
Main gene ontology and pathway clusters revealed by enrichment analysis of genes identified in the genetic studies of Behçet's disease. Both annotated genes (*bold* font) and candidate genes identified by functional enrichment and gene-gene interaction in this study (*regular* font) listed in Table 2 were included in the analysis. Only terms with *p*-value < 1 × 10^−3^ and at least three genes were considered.

Finally, we performed a pathway enrichment analysis to yield new insights into the biological processes that might be relevant in the pathogenesis of Behçet's disease. The identification of key pathways was performed using Metascape (44) by analyzing both the annotated and candidate genes (Table 2). The Gene Ontology (GO) Biological Process branch and Kyoto Encyclopedia of Genes and Genomes (KEGG) pathways were selected and only terms with *p*-value < 1 × 10^−3^ and at least three genes were considered. Bonferroni corrected *p*-values (q values) were calculated as well. A detail list of all results, including genes and *p*-values are displayed in Supplementary Table 3. Since ontologies are often redundant, Metascape categorized the results using a cluster-based approach to simplify their interpretation and to avoid reporting overlapping terms. 13 different clusters were significantly enriched (corrected *p*-values < 0.05): positive regulation of interferon-gamma production (*p*-value = 2.42 × 10^−11^), regulation of lymphocyte mediated immunity (*p*-value = 4.45 × 10^−10^), inflammatory bowel disease (*p*-value = 6.38 × 10^−10^), cytokine-cytokine receptor interaction (*p*-value = 9.98 × 10^−9^), regulation of innate immune response (*p*-value = 1.35 × 10^−7^), regulation of interleukin-12 production (*p*-value = 7.20 × 10^−6^), cell chemotaxis (*p*-value = 1.38 × 10^−4^), cellular defense response (*p*-value = 2.56 × 10^−4^), negative regulation of leukocyte activation (*p*-value = 1.55 × 10^−3^), positive regulation of JNK cascade (*p*-value = 7.34 × 10^−03^), regulation of osteoclast differentiation (*p*-value = 1.49 × 10^−2^), positive regulation of gliogenesis (*p*-value = 2.09 × 10^−2^), and response to virus (*p*-value = 2.31 × 10^−2^). The top clusters, including genes within each cluster, are represented in Figure 2. These results are in line with the hypothesis that Behçet's disease can result from a disruption of the immune system after an infectious trigger. These results also highlight the close genetic relationship between Behçet's disease and inflammatory bowel disease (45).

## Missing Heritability

Despite the considerable advances made in our understanding of the genetics underlying Behçet's disease, it is believed that additional susceptibility factors remain unidentified. However, the complete role of genetics in Behçet's disease is unclear as no accurate estimation of the relative contribution of genetic factors vs. non-genetic factors in this disease has been reported.

Familial aggregation of Behçet's disease has been reported repeatedly showing differences among populations (46–48). In addition, a higher familial incidence was also observed in juvenile patients with the disease compared with patients diagnosed during adulthood (49). One approach to measure the magnitude of genetic factors in complex disease is to calculate the sibling recurrence risk ratio (λs), which is defined as the ratio of the risk of being affected among the siblings of patients and the risk of being affected in the general population. In this sense, Gul and collaborators reported a λs of 11.4–52.5 for Behçet's disease in Turkish population (50). In addition, a higher concordance rate of monozygotic twins compared to dizygotic twins was suggested in Turkish population (51). All these data support the important role of genetic factors in the pathogenesis of Behçet's disease, however, they do not clarify what portion of the disease is attributable to genetic factors and how many susceptibility loci might exist.

Heritability is a population parameter that can be defined in broad-sense (H^2^) as the proportion of total phenotypic variation among individuals that can be attributed to genetic factors. This includes additive and non-additive genetic effects such as interaction between alleles on the same loci (dominance) or at different genetic loci (epistasis). Heritability in a narrow-sense (h^2^) estimates the genetic component of a phenotype that is due to additive genetic variance, and therefore, does not include possible genetic interactions (52). New approaches have been developed recently to use genome-scale SNPs data to estimate heritability in unpedigreed populations. These methods are based on the assumption that a sample of unrelated individuals is unlikely to be confounded by common environmental factors and, therefore, phenotypic similarity should correlate with genetic similarity (53). In this context, the genomic era has brought new insights into the heritability of multitude of complex human traits.

Here we aimed to estimate, the heritability of Behçet's disease for the first time. We used data from a large case-control cohort of Turkish population including a total of 2,344 Behçet's disease patients and 1,920 unrelated healthy individuals genotyped or imputed to include genetic data on loci represented on the Immunochip array (24). We applied the GREML-LDMS method implemented in the genome-wide complex trait analysis (GCTA) software (54, 55). To control for population stratification, we included the first five ancestry principal components as covariates in the model. We found that Behçet's disease heritability in narrow-sense (h^2^) on the observed scale, which is the proportion of variance in case-control status (0 or 1), was 0.36 [Standard Error (SE) = 0.03] in our Turkish dataset. Since it is usual that the proportion of cases included in genetic studies is higher than the general prevalence of the disease, the software implements the option to correct for the ascertainment bias and the observed scale heritability can be transformed to a liability scale (56). The advantage of working on a liability scale is that heritability values can be compared across traits or populations. The proportion of cases in our cohort was 55% and a prevalence of 420/100,000 of Behçet's disease in Turkey has been reported (57). Our estimates for Behçet's disease liability reached 0.16 (SE = 0.02). According to the statistical power calculator implemented in GCTA (58), the probability of estimating heritability on a liability scale of ≥ 0.1 in our cohort was >0.83.

We then estimated the heritability of Behçet's disease explained by the genetic risk loci identified to date in this disease. We selected all SNPs included within ± 1 Mb of the lead SNP of each Behçet's disease-associated locus, as previously described (59). Unfortunately, no SNPs within the *TFCP2L1* and *RIPK2* associated loci were available in our dataset. Our analysis revealed that the estimated heritability of the disease using the known associated genetic regions in Behçet's disease was 0.20 (SE = 0.02) on the observed scale and 0.094 (SE = 0.01) on the liability scale. These data suggest that the total variance explained by the currently known genetic susceptibility loci for Behçet's disease accounts for 58.75% of the genetic contribution to the development of Behçet's disease. This implies that ~40% of the genetic etiology of Behçet's disease remains to be identified.

It should be noted that the SNP-based methods to estimate heritability provide a lower bound of h^2^ since other genetic factors that influence h^2^, such as copy number variation and insertion/deletions, are not considered. In addition, it is also worth to highlight that heritability values we obtained are minimum values of suggested heritability as they reflect the immune-related loci included on the Immunochip. In addition, we did not have data from other populations sufficiently powered to estimate heritability, and despite that heritability values are often similar between populations, our heritability estimation should not be used to predict heritability in other populations.

## Cumulative Genetic Risk Score

The genetic risk score (GRS) is a simple and intuitive approach used to estimate the genetic propensity to a trait at the individual level. It is calculated by summing risk alleles corresponding to a phenotype of interest, with each allele weighted by its effect size estimated from an independent GWAS in the phenotype. Here, we aimed to calculate the genetic risk score of healthy individuals from different populations to check if genetic variance in the loci associated with Behçet's disease correlate with the differences in the prevalence of the disease.

We followed the method described by Hughes et al. to calculate a cumulative genetic risk score (60). The lead SNP in each locus was included in the model. When a lead SNP is not clear, the most significant SNP in a given locus in the largest single cohort reported was used. In case of a clear evidence for multiple independent associations within the same genetic region, all the independent signals were represented. Association odds ratios were derived from published data in Behçet's disease (Supplementary Table 2). We examined the GRS of a total of 2,504 individuals from the five major populations included in the 1000 Genomes Project phase 3 release: African, *n* = 661; Admixed American, *n* = 347; East Asian, *n* = 504; European, *n* = 503; South Asian, *n* = 489 (61). To determine if the cumulate GRS was different across populations, one-way ANOVA followed by Tukey's multiple comparison test was performed using GraphPad Prism version 8.1.1 (GraphPad Software, La Jolla California USA). ANOVA *p*-values < 0.05 and Tukey's adjusted *p*-values < 0.05 were considered significant.

**Figure 3:**
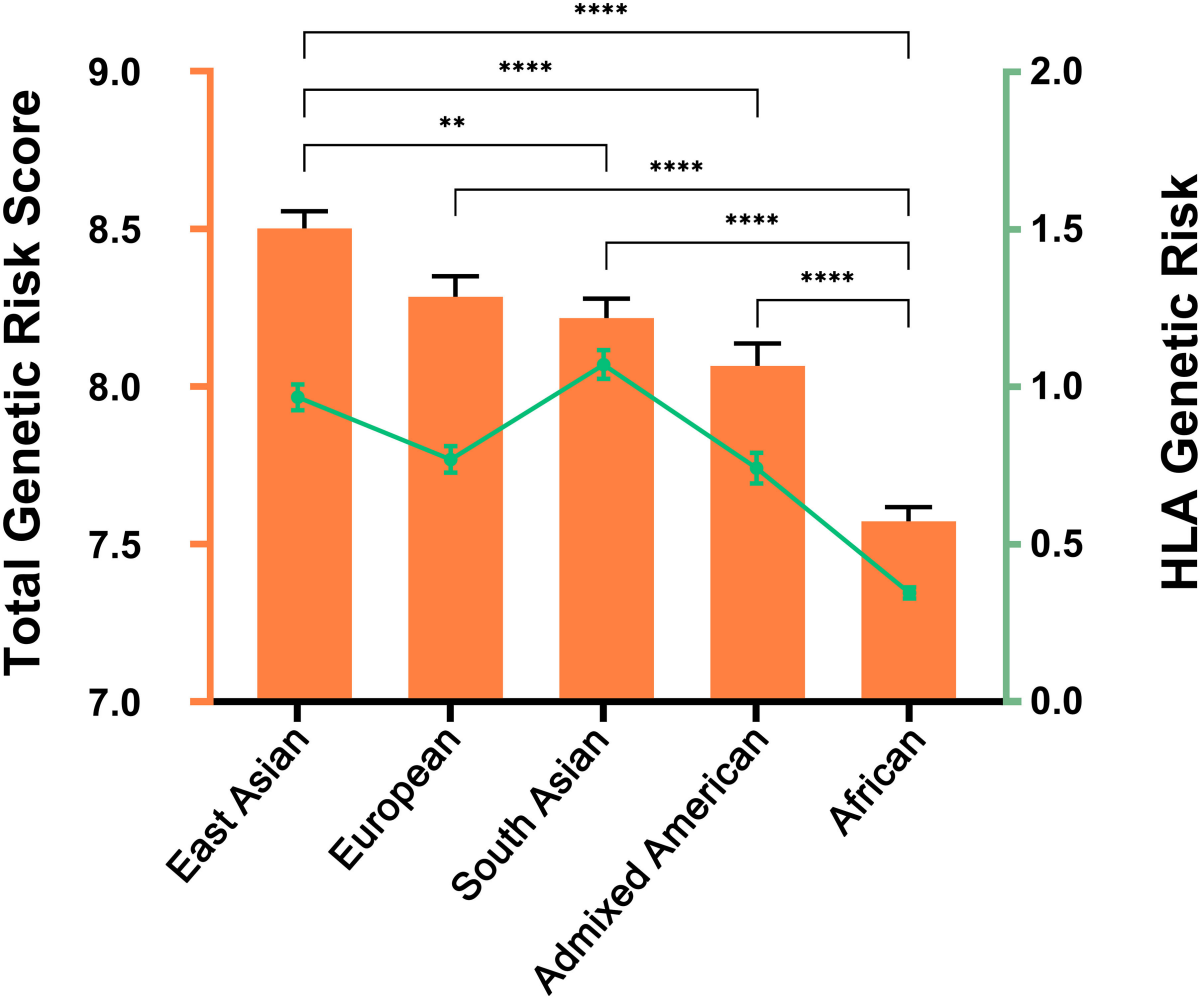
Genetic Risk Score (GRS) for Behçet's disease across the five major populations included in the 1000 Genomes Project. Data were used from the 1000 Genomes Project, phase 3 release. African, *n* = 661; Admixed American, *n* = 347; East Asian, *n* = 504; European, *n* = 503; South Asian, *n* = 489. The orange color illustrates the total GRS including all the genetic susceptibility loci. Columns represent the mean and standard error of mean (error bars) for each population. One-way ANOVA *p*-value was <0.0001 for both total GRS and for HLA genetic risk. Pair-wise differences were evaluated using Tukey's multiple comparisons tests and *p*-values depicted represent analysis using total GRS. **, *P* < 0.01; ****, *P* < 0.0001. The green color corresponds with the GRS considering only the HLA region. Mean and standard error of mean (error bars) are depicted for each population.

The results obtained were in line with the prevalence data that have been reported in Behçet's disease (4, 5). Our results revealed significant differences between the genetic risk scores among populations (ANOVA *p*-value < 0.0001). The highest GRS was observed in East Asian, followed by European, and South Asian populations, while Admixed Americans and African populations showed the lowest values (Figure 3). In addition, we further used the same approach to calculate the GRS considering only the HLA genetic associations to test the contribution of the HLA in the ancestral differences identified. Overall, the genetic risk within the HLA region appears to trend along the total genetic risk for Behçet's disease across populations, with the highest HLA genetic risk detected in Asian populations and the lowest in African populations. Notably, the highest genetic risk arising from the HLA region was detected in South Asian populations (Figure 3). These data support the role of genetics in contributing to the differences in the prevalence of Behçet's disease across populations.

## Future Perspectives and Challenges

Large-scale genetic studies have resulted in a significantly better understanding of the genetic predisposition to Behçet's disease, however, additional genetic susceptibility loci remain to be identified. The lack of large sample sizes to detect disease-associations with low to medium effects remains a limitation. Increasing the sample size in genetics studies has been shown to be effective in other complex diseases (62). However, in diseases with low prevalence such as Behçet's disease, this represents an enormous challenge and huge collaborative international efforts are needed. In this context, another important consideration is the development of well-powered patient cohorts from diverse populations. As in most of the complex traits, there is an under-representation of participants from diverse populations in Behçet's disease genetic studies (63). The inclusion of diverse populations and transancestral mapping have been shown helpful to pinpoint causal genetic variants in complex diseases.

It is perhaps not too distant in the future that data obtained from large-scale genetic studies can be routinely applied to health care of individual patients. Nevertheless, there is still much to be learned about the genetics of Behçet's disease and, importantly, how the associated genetic variants lead to pathogenic consequences predisposing to the disease. Novel insights can be gathered by applying new analysis methods to existing genetic data as we have demonstrated in this publication. For example, the integration of genetic data with other large functional datasets, such as epigenetic features and gene expression datasets, among others, can help to decipher which cell types are relevant for the disease. In this sense, further functional studies can be prioritized to relevant cell types involved to elucidate the molecular effects of disease-associated genetic variants. A complete understanding of the genetic contribution to the development of Behçet's disease, the pathways and cell types involved, and the resulting functional disturbances, will help to achieve better management strategies in this complex disease.

## Author Contributions

All authors wrote and critically revised the manuscript.

## Conflict of Interest

The authors declare that the research was conducted in the absence of any commercial or financial relationships that could be construed as a potential conflict of interest.
